# Commentary: Establishment of reference intervals for complete blood count in healthy adults at different altitudes on the Western Sichuan Plateau

**DOI:** 10.3389/fmed.2025.1642003

**Published:** 2025-07-09

**Authors:** Mohammed Abdulrasak, Ali M. Someili, Mostafa Mohrag

**Affiliations:** ^1^Department of Clinical Sciences, Lund University, Malmö, Sweden; ^2^Department of Gastroenterology and Nutrition, Skåne University Hospital, Malmö, Sweden; ^3^Department of Medicine, Faculty of Medicine, Jazan University, Jazan, Saudi Arabia

**Keywords:** hematologic reference intervals, population genetics, regulatory variants, precision medicine, global health diagnostics

We read with great interest the recent article by Wang et al. (1) in *Frontiers in Medicine* describing hematologic reference intervals among healthy adults living at different altitudes on the Western Sichuan Plateau. Their work represents an important step in refining diagnostic thresholds for high-altitude populations and highlights the limitations of applying sea-level or internationally derived laboratory standards to communities with distinct environmental exposures.

While the study focuses primarily on physiological adaptation to chronic hypoxia, we wish to extend the conversation by underscoring the need to integrate ancestral and genetic variation—particularly regulatory and noncoding variants—into such efforts (2). Population-specific diagnostics should eventually account not only for environmental modifiers like altitude but also for inherited traits that shape baseline hematologic profiles and affect disease susceptibility or therapeutic response (3).

For example, noncoding erythropoietin (EPO) promoter variants recently linked to hereditary erythrocytosis suggest that population-enriched regulatory mutations can elevate red cell production independent of serum EPO levels detectable by standard assays (3, 4). Similarly, benign ethnic neutropenia (BEN), mediated by the Duffy-null genotype (5), remains a classic illustration of how genetically influenced baselines can be misread as pathological when majority-derived reference ranges are applied (6). Moreover, recent work demonstrating the therapeutic potential of Cas9-mediated insertion of natural EPO variants points to a future where regulatory polymorphisms may not only inform diagnosis but also offer treatment options for hematologic conditions (7).

We acknowledge that integrating genetic and glycomic analyses into routine diagnostics poses cost and logistical challenges, particularly in lower-resource settings and rural or high-altitude communities (8). However, we believe that investments in scalable, context-sensitive genomics, coupled with environmental adaptation studies like that of Wang et al., will be crucial to building inclusive, precise, and globally relevant diagnostic frameworks.

We commend the authors for their contribution to hematologic equity and encourage further research at the intersection of ancestry, environment, and regulation to redefine what “normal” means in diverse populations.
